# Mechanisms to Avoid and Correct Erroneous Kinetochore-Microtubule Attachments

**DOI:** 10.3390/biology6010001

**Published:** 2017-01-05

**Authors:** Michael A. Lampson, Ekaterina L. Grishchuk

**Affiliations:** 1 Department of Biology, University of Pennsylvania, Philadelphia, PA 19104, USA; 2 Department of Physiology, Perelman School of Medicine, University of Pennsylvania, Philadelphia, PA 19104, USA

**Keywords:** Aurora B kinase, kinetochore geometry, microtubule turnover, tension-dependent regulation

## Abstract

In dividing vertebrate cells multiple microtubules must connect to mitotic kinetochores in a highly stereotypical manner, with each sister kinetochore forming microtubule attachments to only one spindle pole. The exact sequence of events by which this goal is achieved varies considerably from cell to cell because of the variable locations of kinetochores and spindle poles, and randomness of initial microtubule attachments. These chance encounters with the kinetochores nonetheless ultimately lead to the desired outcome with high fidelity and in a limited time frame, providing one of the most startling examples of biological self-organization. This chapter discusses mechanisms that contribute to accurate chromosome segregation by helping dividing cells to avoid and resolve improper microtubule attachments.

## 1. Introduction

Segregating chromosomes equally is a non-trivial task, which cells face every time they divide. Healthy human cells have 46 chromosomes, all of which must be duplicated and then segregated equally [1,2]. If segregation fails, the daughter cells may acquire an inappropriate number of chromosomes (aneuploidy), which is associated with severe developmental abnormalities and diseases, including cancer [3,4,5,6]. The correct outcome is achieved reproducibly and with high accuracy despite a large stochasticity and variability in many parameters: number and size of the chromosomes, their initial locations, and stochastic behavior of spindle microtubules (MTs), to name a few. MTs originating from the pole can be captured by the kinetochore from almost any angle [7]. Subsequent kinetochore motions, translational and rotational, bring it in contact with more MTs. A human kinetochore ultimately binds 20 MTs on average [8,9], but the real task is to ensure that all these MTs are to one pole, while the MTs at the sister kinetochore are connected to the opposite pole, referred to as the amphitelic configuration. If MTs from two poles attach to sister kinetochores completely randomly, the probability of finding such a configuration is less than 10^−10^. The accuracy of segregation is orders of magnitude better, although the exact frequency of chromosome loss in somatic human cells in vivo is not well known. In normal cells, the mis-segregation rate during one division lies in the range 10^−4^–10^−3^ per chromosome [10,11,12], corresponding to one mis-segregating chromosome in 10^2^–10^3^ dividing cells. Immortalized human cell lines, which are typically derived from different tumors, are often aneuploid, and the mis-segregation rate per chromosome is ~10 times higher [13]. Even at this rate, the segregation outcome is impressive and indicates robust self-organization to avoid or resolve MT attachment errors. In addition, a surveillance system buys time if normal mitotic progression is perturbed, as considered in another chapter in this issue by A. Joglekar. Below, we focus on principal mechanisms that resolve MT attachment errors and collectively ensure accurate chromosome segregation.

## 2. Literature Review Sections

### 2.1. Error Correction of Syntelic Attachments

**Figure 1:**
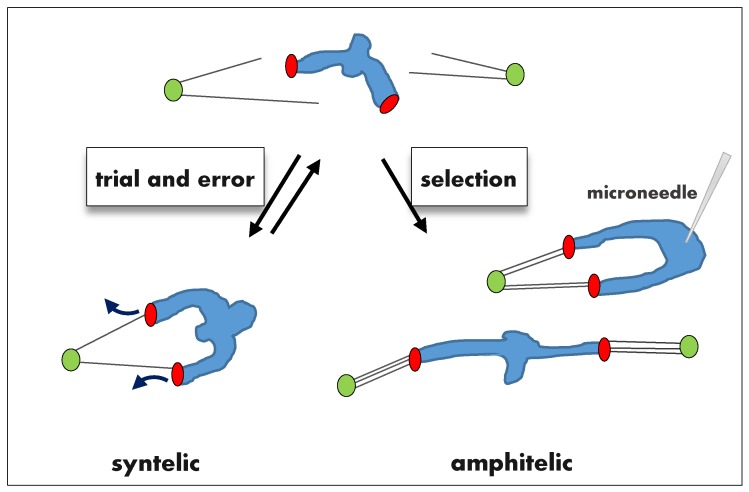
Tension-dependent error correction for syntelic kinetochore microtubule (KMT) attachments. Kinetochores (red) of a meiotic bivalent (blue) repeatedly bind MTs from different poles (green), but KMT attachments in the syntelic configuration are short-lived. Tension arises when the amphitelic configuration is encountered, inducing stable KMT attachments. Syntelic attachments can be stabilized artificially by applying tension with a microneedle. In meiosis I, as shown here, kinetochores of homologous chromosomes attach to opposite spindle poles in the amphitelic orientation. In mitotic cells, kinetochores of sister chromatids would attach to opposite poles.

The conceptual framework for error correction was laid out by B. Nicklas, whose work focused on syntelic attachment errors, in which kinetochores that should be attached to opposite poles are instead attached to the same spindle pole, in meiosis I (Figure 1). Error correction for this type of attachment results from Darwinian selection of the amphitelic kinetochore MT (KMT) configuration (reviewed in [7]). The basis for this mechanism is that KMT attachments are inherently unstable, so errors can be corrected via trial and error, as MTs are released from kinetochores and new ones are captured (called KMT turnover). Nicklas suggested that correction of syntelic attachments involves repeated cycles of complete detachment and reattachment of KMTs until the correct, amphitelic configuration is encountered. As for any selection process, a feedback is required to reinforce the desired outcome. Nicklas’s work established the dominant idea in the field that this feedback is provided by tension exerted by spindle MTs pulling sister kinetochores in opposite directions. Because of the spindle geometry, tension is higher for amphitelic attachments than for incorrect attachments, so these attachments can be discriminated from each other. Classic experiments in grasshopper spermatocytes [14] provided evidence supporting this idea: syntelic attachments are unstable unless tension is applied with a micromanipulation needle by pulling the chromosomes away from the attached pole (Figure 1).

Analogous manipulations in vertebrate tissue culture cells have proven difficult [15], so a direct demonstration of tension-induced stabilization of KMT attachments during somatic cell division is lacking. In a molecular variation of Nicklas’s experiment, tension was applied in mitotic Drosophila cells by overexpression of a chromokinesin, a plus-end-directed MT motor that localizes to chromosome arms [16]. Increasing chromokinesin levels leads to increased polar ejection forces acting on chromosomes during mitosis. In cells with monopolar chromosomes, these forces increase tension on syntelic attachments and stabilize them. These complementary approaches and other correlative observations provide compelling evidence for tension-dependent stabilization of kinetochore-MT attachments both in mitosis and in meiosis I. At a molecular level this “catch bond” behavior is unusual because increasing tension usually breaks a bond, increasing its dissociation under increasing force. The catch bond will also eventually break if the force is too large, but at intermediate tension this bond is strengthened. Interestingly, Nicklas thought that tension was likely to stabilize the anchorage of MTs at the pole [7]. Current thinking for mitosis is that the tension-modulated bonds are located at the kinetochore, as discussed later in this chapter, although the exact mechanism could vary in different organisms. 

### 2.2. Error Correction of Merotelic Attachments

**Figure 2:**
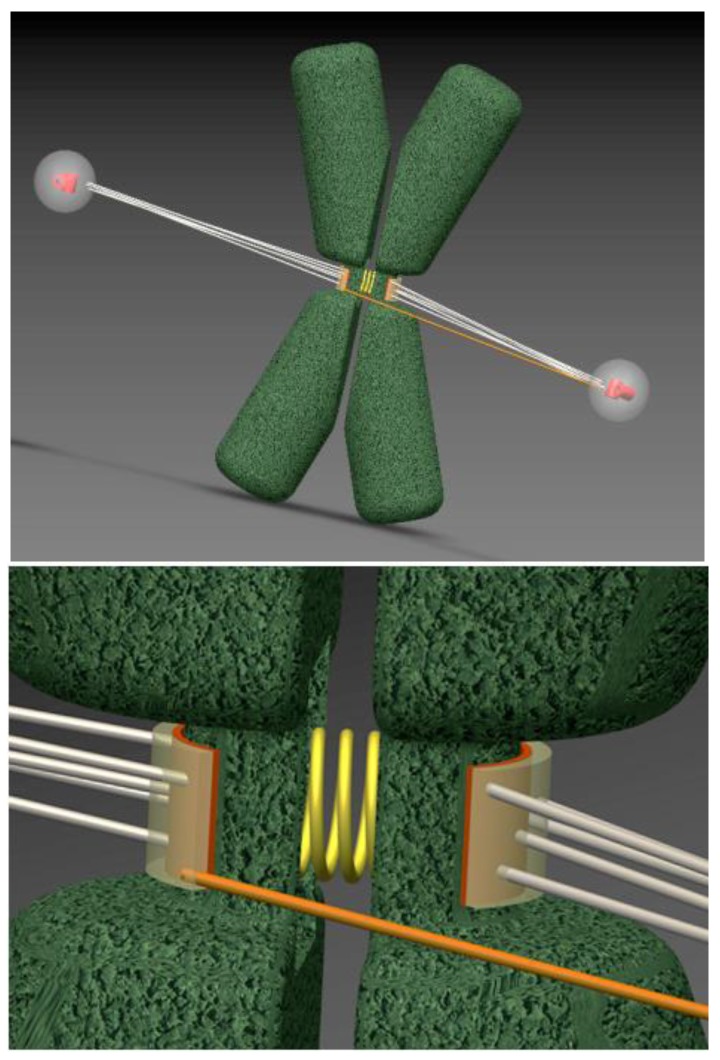
Merotelic KMT configuration for a congressed chromosome. Top image is a three-dimensional representation of a mammalian chromosome (green) positioned midway between two spindle poles. Sister chromatids (green) are connected by a stretchable centromere, represented as a spring (yellow). Bottom image is an enlargement of sister kinetochores, depicted as semi-transparent layers. Most of the attached MTs are in the proper amphitelic configuration (grey), extending from opposite spindle poles. However, although the chromosome has congressed and its sister kinetochores are positioned back-to-back, they can still bind improper merotelic MTs (one such MT is shown in orange). Computer-generated images are snapshots from video material in [23].

In mammalian cells, multiple MTs interact with each kinetochore, and it is difficult to visualize these individual attachments in real time. Although quantitative information is generally lacking, syntelic attachments seem to form rarely during a typical mitosis in human cells [17]. A more common error is merotelic attachment: connection of one sister kinetochore to MTs from both poles, (Figure 2). Both syntelic and merotelic attachments can lead to chromosome segregation errors. Interestingly, cells typically delay anaphase in the presence of syntelic attachments, but merotelic attachments are more dangerous because anaphase can start prior to correcting them [17]. As a result, MTs attached to the wrong pole impede chromosome motion toward the correct pole, leading to lagging chromosomes in anaphase [18,19]. Such chromosomes have been suggested to constitute the most common pathway to create aneuploidy in cancer cells [13,20,21,22]. It is counter-intuitive that the more frequent MT attachment errors are not monitored by a checkpoint, which suggests that normal cell division mechanisms are sufficient for coping with these errors. Little is known about these mechanisms, however, because merotelic attachments are difficult to study without introducing large perturbations.

**Figure 3:**
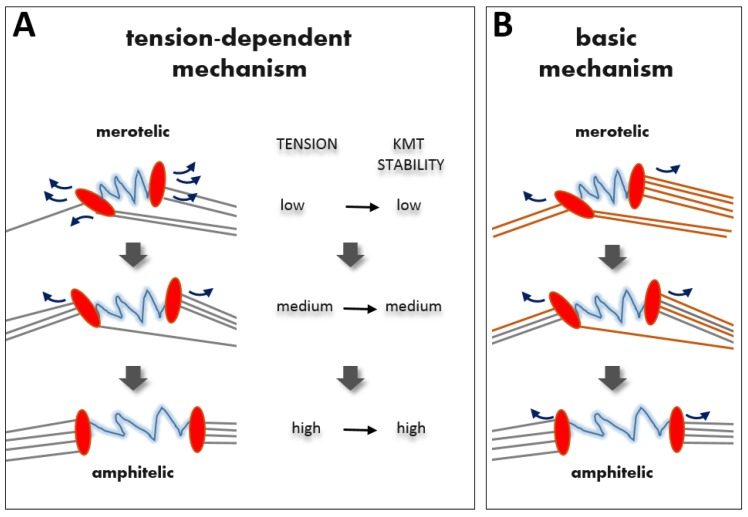
Models for correction of merotelic KMT attachment errors. Correction of merotelic attachments in vertebrate cells proceeds gradually during prometaphase and metaphase without necessarily losing all KMT attachments. (**A**) In the tension-dependent error-correction model, stretching between sister kinetochores (red ovals connected with blue springs) is the primary signal that modulates the turnover of KMT attachments (depicted with curved arrows). As kinetochores bind and lose KMTs, probing different configurations, the configurations that produce higher tension are assumed to induce higher KMT stability. Because their KMTs detach less frequently, such configurations last longer. This gradual evolution would lead eventually to the completely amphitelic configuration, which generates maximal tension. In the non-discriminate version of this model (as shown), all KMTs are affected similarly. Alternatively, in the selective version of this model, lifetime of merotelic vs. amphitelic KMTs is assumed to be regulated differently (see text for details). (**B**) In the basic mechanism, the gradual correction of merotelic attachments proceeds with no change in the turnover of KMT attachments. The rate of KMT turnover in this model does not depend on tension and is not selective, so all old KMTs (orange) eventually detach, whether correct and incorrect, and are replaced with new KMTs (grey). New KMTs preferentially attach correctly, favored by the back-to-back geometry of sister kinetochores.

The most straightforward hypothesis for merotelic attachments is that they are corrected based on the same principle mechanism as syntelic attachments, but direct application of the trial-and-error mechanism to merotelic attachment is problematic. Indeed, for syntelic error correction, the correct configuration can be selected if the response to high and low tension is simply “on” and “off” (Figure 1), so the kinetochore cycles between states with attached MTs and with complete detachment, until finding the configuration that generates tension. Merotelic attachments may also experience a dramatic loss of tension if the number of incorrect MTs is relatively large, in which case a complete KMT detachment may take place. However, cycles of complete kinetochore detachment are not a common feature of somatic mitosis, so merotelic errors appear to improve gradually over the course of prometaphase and metaphase [24]. It is still possible, of course, to view this process as tension-dependent Darwinian selection of the correct KMT configuration by assuming that tension gradually increases with more amphitelic KMTs, leading to slower detachment of MTs from amphitelic kinetochores and faster from merotelic (Figure 3A). This assumption is reasonable if all MTs, correct or incorrect, generate pulling force. Because these MTs pull in different directions, tension between sister kinetochores can potentially serve as a readout for the number of properly attached KMTs. Since the number of amphitelic KMTs improves gradually during a typical mitosis, tension should also modulate KMT attachment stability gradually, guiding the evolution of KMT configurations. In this framework, tension-dependent regulation at a single kinetochore pair is indiscriminate, i.e., it stabilizes all KMTs to the same extent, regardless of whether they are correct or incorrect.

This general concept is appealing but raises many interesting and still unanswered questions. For example, are two sister kinetochores affected by this mechanism equally? As noted above, if the tension between two sisters is the main regulator, the degree of destabilization of KMT attachments should be the same for both kinetochores, even though one of them may have already acquired the correct set and destabilizing this successful outcome would be counterproductive. Also counterproductive would be destabilization of amphitelically attached kinetochores with a reduced number of KMTs, which is normally quite variable [9]. The required sensitivity of such a tension-dependent mechanism also raises some concerns. For example, is it feasible for such gradual regulation to discriminate and correct a single merotelic MT? One improperly attached KMT out of 20 is expected to reduce tension at the kinetochore by only 5%, which is less than the normal variation in inter-kinetochore tension associated with directional chromosome instability during metaphase oscillations. The overall speed of this regulatory mechanism, which is inherently limited by the rate of KMT turnover, may also become an issue, because during normal mitotic duration the kinetochores would have to sort through a large number of wrong KMT configurations, the essence of the Darwinian selection mechanism. In general, designing a robust regulatory mechanism that senses a small change (1 KMT) for a large range of input signals (from 1 to 20 KMTs), while quickly inducing a strong (stabilizing) response after all KMTs have attached properly, is not trivial, so future experiments and theoretical calculations should address the feasibility of the Darwinian regulatory mechanism for merotelic errors.

Finally, it is not clear how these views of error correction can be reconciled with the concept of force balance at metaphase kinetochores [25,26]. Because the tension-dependent stabilization model is concerned only with the magnitude of inter-kinetochore tension, it is often overlooked that this selection mechanism could be accompanied by a large and variable imbalance in the kinetochore pulling forces. Unbalanced forces acting on sister kinetochores result in directed chromosome motion, but large chromosomal excursions are rare during metaphase. Polar ejection forces and kinetochore-localized regulators of KMT dynamics are thought to dampen chromosome oscillations (e.g., [27]). These forces can also affect inter-kinetochore tension, however, so why they do not interfere with the tension-driven error-correction mechanism is unclear.

An interesting possibility is that mammalian cells need not make 100% accurate, amphitelic attachments. Perhaps some degree of merotely can be tolerated because it would lead to a fairly small reduction of the pulling force in anaphase. In the ensuing tug-of-war between properly attached kinetochore MTs and a few MTs pulling in the wrong direction, the main KMT bundle can apparently win [28], leading to normal segregation of a merotelic kinetochore. This strategy seems dangerous, however, because chromosome velocity in anaphase slows down while the wrong attachments are being resolved, thereby delaying arrival of the affected chromosome at the site of nuclear envelope reformation. The late arriving chromosome may end up excluded from the main nucleus, forming its own micronucleus [29]. Normal somatic cells arrest the cell cycle in the presence of such micronuclei, but in cells with p53 mutation, disastrous rearrangement of DNA in a micronucleus may take place, contributing to tumorigenesis [30,31]. Nonetheless, this back-up mechanism for correcting merotelic attachments during anaphase segregation may play an important role when the number of merotelic KMTs is small, relieving the difficult requirement for high sensitivity of the error-correction mechanism. It is also interesting in this respect that the rate of chromosome mis-segregation reported for mitotic human cells is higher than in yeast: ~10-fold when adjusted for chromosome number [12]. The reasons for this difference are unclear but could indicate more relaxed requirements for fidelity of chromosome segregation in human cells.

### 2.3. Tension-Independent Error-Correction Mechanism

Although tension-dependent MT stabilization traditionally receives major attention for its role in error correction, other factors are known to play a significant role. In particular, the specific geometry of vertebrate kinetochores is an important contributor to the accuracy of mitotic chromosome segregation [32]. It has long been recognized that the back-to-back arrangement of sister kinetochores creates geometric constraints which favor sister kinetochore attachment to opposite spindle poles [33]. Little is known, however, about the stringency of these constraints and the exact role they play in mitosis. In the traditional view of Darwinian selection via tension-dependent stabilization, this geometry does not help to correct the already formed merotelic MTs but rather reduces the number of initial wrong attachments [7].

The alternative view, which is not incompatible with the first one, is that geometric constraints constitute an integral part of a simplified error-correction mechanism that does not rely on tension-dependent stabilization, but incorporates KMT turnover in addition to restrictive kinetochore geometry (Figure 3B). This mechanism can be understood intuitively assuming that improper kinetochore MTs are acquired during early stages of mitosis, and that their subsequent release is the rate-limiting step for successful bi-orientation [34]. Indeed, after the kinetochores become positioned favorably (i.e. midway between spindle poles), all bound KMTs will be gradually released due to turnover. Stringent geometric constraints, however, will favor their replacement only with proper, amphitelic MTs. This mechanism is feasible because the turnover time for KMTs in human cells is 2–6 min [35,36,37]. With this release rate, all KMTs that attached to kinetochores earlier in mitosis will be replaced during the normal duration of metaphase (10–20 min). Interestingly, prolonging mitosis appears to elicit a relatively small improvement in error correction (2.7-fold decrease in lagging chromosomes) [24]. This finding suggests that the normal mitotic clock closely matches the kinetics of KMT turnover. A combination of these matching kinetics, the back-up mechanism to resolve small numbers of merotelic KMTs in anaphase and the overall segregation accuracy that can be tolerated, may explain why vertebrate cells do not utilize a checkpoint to buy more time to achieve perfect amphitelic KMT attachments. 

The positive effect from this tension-independent error-correction mechanism is maximal when the kinetochore pair is positioned favorably. Thus, it should benefit strongly from expedient chromosome congression, because achieving a position midway between the poles marks the start of a productive time toward the steady-state configuration with a minimal number of merotelic KMTs. Consistent with this view, chromosomes can congress via different mechanisms and even without bi-orientation [38], enabling them to assume a midway position quickly. Interestingly, for successful operation of this error-correction mechanism, it is not necessary to assume that the incorrect KMTs are less stable than the correct ones, so in this sense regulation of KMT stability is indiscriminate [39]. Calculations suggest that if small numbers of merotelic MTs can be tolerated, a combination of indiscriminate KMT turnover and geometric constraints can enable normal segregation of 45 out of 46 chromosomes during cell division [23]. This fidelity is less than physiological but is a vast improvement over completely random attachments, so it is appropriate to call the underlying processes the “basic” error-correction mechanism. In the next two sections we discuss in more detail the specific roles played by kinetochore geometry and KMT turnover in ensuring accurate chromosome segregation within the framework of the basic mechanism. 

#### 2.3.1. How Strongly Should Kinetochores Shield Themselves from Wrong MTs?

**Figure 4:**
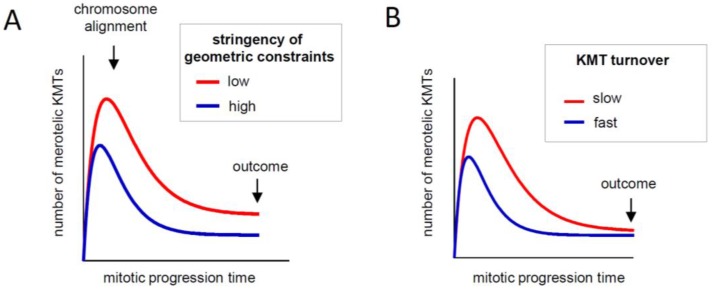
Quantitative analysis of the evolution of KMT configurations during mitotic progression. Graphs illustrate changes in the number of merotelic KMTs during mitotic progression, as predicted by the basic error-correction model [23]. After mitosis starts, the number of attached MTs, some of which are merotelic, increases sharply. As chromosomes become aligned midway on the spindle, the number of merotelic KMTs begins to decline. However, merotelic KMTs are not eliminated completely by this mechanism because new erroneous attachments continue to form even on congressed chromosomes, albeit at lower frequency. Thus, the system tends toward a steady state, in which the rates of forming wrong attachments and eliminating them through non-discriminatory turnover are balanced. (**A**) With more stringent geometric constraints, the final outcome for the KMT configuration at anaphase onset is improved because the rate of capturing wrong KMTs at steady-state is reduced. (**B**) The rate of KMT turnover does not affect the final outcome (black arrow). However, if anaphase starts before the steady state is reached, cells with slower KMT turnover will have more merotelic KMTs.

The stringency of geometric constraints appears to be different at different mitotic stages. Since the basic mechanism is most productive after the chromosomes have congressed, shielding them from wrong MTs at this stage is critical. In dividing mammalian cells, however, the binding of inappropriate MTs to metaphase kinetochores is still possible [24]. Various factors could explain this observation. For example, the spindle structure is not linear, so not all kinetochores can become aligned perfectly along its axis. It is also not completely static: the entire spindle moves within the cell, the poles move relative to one another, and chromosomes are subjected to various forces, including thermal. The centromeric material is relatively elastic, allowing sister kinetochores to deviate from perfect back-to-back orientation. Together, the resulting motions could permit capture of inappropriate MTs even on aligned and oriented sister kinetochores. Calculations show that the frequency of such attachments will directly affect the number of merotelic KMTs at anaphase onset (Figure 4A). Thus, any mechanical or structural feature that stabilizes the co-alignment of the kinetochore pair and spindle axes would help to optimize operation of the basic mechanism and improve the accuracy of segregation. In this sense, the entire spindle and its mechanical properties constitute an important factor in mitotic error correction, explaining why segregation accuracy can be reduced via so many different molecular perturbations.

Geometric constraints during earlier mitotic stages appear to be much less stringent. In mammalian cells, ~10% of chromosomes have sister kinetochores in a side-by-side orientation [40], which is highly permissive to the formation of multiple merotelic attachments. During prometaphase the relaxed geometric constraints may be advantageous because they increase the probability of MT capture. Calculations show that the traditional “search and capture” mechanism with interphase MT dynamics is inefficient [41]. Mitotic cells speed up this search by increasing MT dynamics, but some studies suggest that capture is still problematic in a crowded environment with a large number of chromosomes [42,43]. If kinetochore geometry during prometaphase was as restrictive as is desirable for metaphase kinetochores, the capture of MTs early in mitosis would be greatly delayed [23], so the frequency of MT capture by kinetochores is likely to be a limiting factor for mitotic progression. It appears that prometaphase kinetochores overcome this limitation by enlarging their coronas and increasing curvature, despite the elevated risk of binding wrong MTs [44,45,46]. Little is known, however, about the underlying molecular mechanisms and the processes by which kinetochores shed coronas and become more compact during metaphase, when the capture of inappropriate MTs must be avoided.

#### 2.3.2. What Determines the Rate of KMT Turnover during Mitosis?

KMT turnover is central for error correction in both tension-dependent and tension-independent models, but the degree of KMT stabilization at amphitelic kinetochores is often overstated. The KMT half-life time in many human cells increases from 2–3 min in prometaphase to 4–6 min in metaphase, indicating not more than 2–3-fold stabilization [35,36,37]. The tension-dependent model explains this effect by evolution of KMT configurations from merotelic to amphitelic. Interestingly, however, careful measurements of KMT turnover in perturbed mitotic cells suggest that turnover decreases later in mitosis owing to a timing mechanism, which is not dependent on achieving the amphitelic configuration [36].

Error correction via the basic mechanism does not require that KMT turnover slows down in metaphase, but other aspects of mitotic physiology could explain the constrained kinetics. As discussed earlier in this review, a lower limit on the KMT turnover rate is likely to be imposed by the normal duration of metaphase, because if anaphase starts before all KMTs have been replaced with new ones, some wrong KMTs that could have been removed will remain and lead to lagging chromosomes [19] (Figure 4B). This logic suggests that faster turnover should increase the rate of error correction and promote higher fidelity of chromosome segregation. Such correlation is indeed observed in different cell lines [21]. Moreover, moderate destabilization of the overly stable kinetochore MTs in cancer cell lines improves the accuracy of segregation (reviewed in [47]). Consistently, normal cells do not tolerate induced MT stabilization, as it leads to chromosome segregation errors.

**Figure 5:**
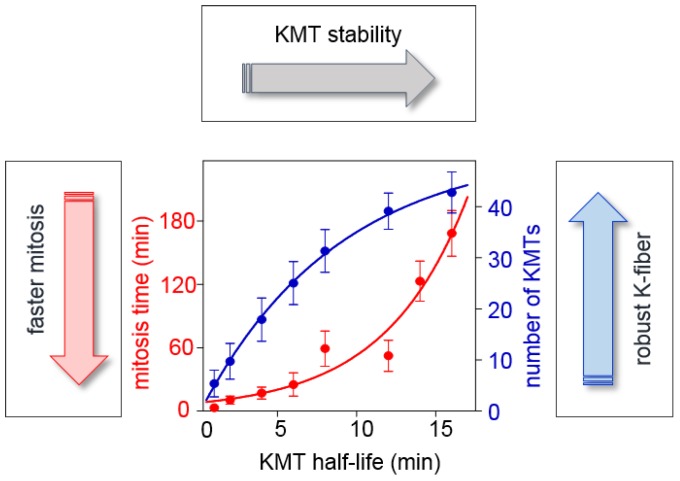
Competing constraints on KMT turnover during mitotic progression. Graph illustrates how KMT turnover (represented as KMT half-life, horizontal axis) affects the overall speed of mitosis and the total number of acquired KMTs (based on the error-correction model in [23]). The speed of mitotic progression (left axis) is evaluated based on the time required to achieve the steady-state KMT configuration. At steady state, the number of attached KMTs (right axis) and the fraction of merotelic KMTs (not shown) have stopped changing, and increasing the duration of mitosis does not generate more KMTs or improve accuracy. When KMT turnover is slow, i.e., KMT half-life is longer, attachments are more stable because they have low release rate. The time required to release all old KMTs increases quickly with increasing KMT stability (red curve), so shorter KMT lifetime is required for speedy mitotic progression. However, the number of KMTs in a K-fiber is lower for shorter KMT lifetime (blue curve). Thus, the limited time of mitotic progression and the acquisition of a full set of KMTs represent the competing constraints on the rate of KMT turnover during mitosis.

These results make sense in the framework of the basic mechanism because merotelic errors are not detected and metaphase duration is limited, so cells with slower turnover should have more lagging chromosomes when anaphase starts. But why then do normal dividing cells slow down their rate of KMT turnover in metaphase, opposite to what this logic suggests? With no such retardation, anaphase could start earlier without compromising the accuracy of this mechanism (Figure 5, red curve). The answer may lie in the physiological requirement to form a robust kinetochore fiber, a process that depends strongly on KMT turnover and may impose an upper limit on its rate. Calculations show that a fully sized kinetochore fiber cannot form if KMTs exchange too quickly, regardless of how long the cell waits [23]. Building a kinetochore fiber with 20-25 KMTs, is problematic if the KMT half-life is 2–3 min, as in prometaphase (Figure 5, blue curve). To acquire this set, the half-life of KMTs should be increased to 4–6 min. Thus, increasing the overall stability of KMT attachments in metaphase may be a compromise reflecting the multiple roles played by KMT turnover during mitosis and the need to balance accuracy of chromosome segregation, speed of mitosis and acquisition of fully sized KMT fibers [23].

### 2.4. Molecular Mechanisms of Tension-Dependent Feedback for KMT Stabilization

#### 2.4.1. Direct Regulation of MT Dynamics

As described above, the basic error-correction mechanism can potentially provide vast improvement in accuracy over random MT attachments, but the expected mis-segregation rate for this mechanism is still high: 10^−1^–10^−2^. This theoretical prediction is close to the rate in some cancer cell lines, but the mis-segregation rate measured in human RPE-1 cells is much lower: ~10^−4^ [13]. Therefore, additional error-correction mechanisms in normal cells should provide more than 100-fold improvement in the rate of chromosome mis-segregation on top of the basic mechanism. It is likely that such fidelity is achieved by a combination of the basic mechanism and tension-dependent stabilization of proper attachments. While many questions remain about the operation of such a combined mechanism, molecular details of how tension regulates KMT stability, a long-standing goal in the field, are beginning to emerge. 

Tension has been suggested to directly regulate dynamics of kinetochore-bound MT ends because pulling force applied to the depolymerizing MT end slows depolymerization and promotes MT rescue [48,49]. Furthermore, tension of a few piconewtons applied via an optical trap to isolated yeast kinetochores increases the lifetime of MT attachment, although the stability decreases with further increase in force [50]. This bell-shaped dependency suggests the long-sought catch-bond, but apparently it does not result from a specific molecular linkage. Rather, this behavior reflects a property of the MT to switch into polymerization under pulling tension. Since yeast kinetochores bind much more strongly to growing MT plus-ends than to shortening ones, force increases the lifetime of attachment to dynamic MT ends by promoting a longer duration for the MT polymerization state. If this mechanism contributes to tension-sensitive stabilization in vivo, then KMT plus-ends should spend more time in the polymerizing state when they are in an amphitelic configuration than when they have incorrect attachments. This prediction has not been directly tested, but in budding yeast, in which bi-oriented kinetochores oscillate in the absence of poleward MT flux, MT polymerization at one kinetochore is balanced by depolymerization at the sister, so both would not be in the strongly attached state simultaneously. In some organisms poleward MT flux can increase the time spent in the polymerizing state for both sisters, but the velocity of flux in mammalian cells is significantly lower than the rate of chromosome oscillations, so at metaphase the kinetochore-bound MT ends spend approximately equal time in polymerizing and depolymerizing states. The alternating states are confirmed by visualization of an EB (end-binding) protein [51,52], which binds to polymerizing MT ends and shows intermittent localization at oscillating kinetochores. It is not clear if this EB localization pattern changes as merotelic attachments are being corrected on congressed chromosomes, as would be predicted if the direct mechanism played a significant role in error correction. 

#### 2.4.2. Aurora B-Dependent Mechanisms

Several other proposed models feature the mitotic kinase Aurora B as a key regulator of kinetochore-MT interactions. Work in yeast and in vertebrate cells shows that Aurora B phosphorylates kinetochore substrates to regulate MT binding, promotes turnover of KMT attachments, and plays an essential role in accurate chromosome segregation [53,54,55,56,57,58,59,60,61]. Furthermore, phosphorylation of Aurora B substrates at kinetochores is generally inversely proportional to tension, although the MT-binding protein Ndc80 is not fully rephosphorylated after KMT attachments that have already formed are disrupted with nocodazole [62,63,64,65,66,67]. These observations and others (recently reviewed in [68,69]) are consistent with the hypothesis that Aurora B-dependent phosphorylation of kinetochore substrates destabilizes incorrect KMT attachments in the absence of tension. 

These results also imply that Aurora B could be a mediator of tension, but how does it affect KMT stability? Aurora B can destabilize attachments in two ways. The first is by directly promoting detachment of MTs from the kinetochore, as suggested by experiments in vitro [63,70,71,72,73]. Consistent with the in vitro findings, kinetochores in mitotic cells cannot maintain stable attachment to MTs when Aurora B activity is increased by targeting the kinase to kinetochores [62]. Phosphorylation of Aurora B substrates at kinetochores, such as the Ndc80 complex, reduces MT binding affinity (reviewed in [68]), and expression of phosphomimetic mutants of Ndc80 in cells leads to fewer KMTs [74]. Perturbing other Aurora B substrates, such as the MT-binding Dam1 and Ska1 complexes, also destabilizes kinetochore-MT attachments [68]. Second, Aurora B can promote catastrophe and depolymerization of KMTs, as shown in vitro using phosphomimetic mutants of Ndc80 and Dam1 [72,73]. Observations in live cells indicate that at syntelic attachments Aurora B promotes MT depolymerization, rather than immediate detachment. In this case, the syntelic kinetochores are first pulled towards the spindle pole, and detachment occurs subsequently as a result of activity of the related Aurora A kinase, which is enriched at the pole [59,75,76]. 

#### 2.4.3. Spatial Separation Model

Why does Aurora B activity at kinetochores change in response to interkinetochore tension? The spatial separation model, first suggested based on experiments in budding yeast [56], proposes that tension sensing depends on the changing distance between Aurora B, which is enriched at the inner centromere, and its MT-binding substrates at the outer kinetochore. When KMT attachments are amphitelic, tension pulls bi-oriented sister kinetochores away from the inner centromere, separating the kinase from its substrates, thereby reducing phosphorylation. This model is supported by the observations described above, showing that phosphorylation decreases with tension. Furthermore, repositioning Aurora B so that it localizes in close proximity to kinetochore substrates leads to increased phosphorylation and destabilization of KMT attachments [62].

Consistent with this model, multiple observations indicate that perturbing centromere localization of Aurora B disrupts normal regulation of kinetochore-MT attachments. As part of the chromosome passenger complex (CPC), Aurora B is targeted to the inner centromere through binding of the CPC to phosphorylation marks on histones H3 and H2A [77,78,79,80]. Mutation of these phosphorylation sites in fission yeast prevents CPC targeting and leads to severe bi-orientation defects. Moreover, these defects are largely rescued by restoring CPC targeting, e.g., by fusing a CPC component, Survivin, to a chromodomain that binds pericentromeric heterochromatin [80]. In human cells, either mutation of Survivin or inhibition of the Haspin kinase, which is responsible for the H3 phosphorylation mark, prevents normal centromere localization of Aurora B, and leads to defects in the correction of attachment errors [79,81]. The same mutation in chicken Survivin did not affect cell growth, but the error-correction process was not explicitly tested in these cells [82]. Furthermore, preventing CPC targeting to centromeres by mutation of Cdk phosphorylation sites also leads to chromosome bi-orientation defects in both fission yeast and human cells. Again, these defects are rescued by restoring localization of the CPC using a chromodomain fused to Survivin [83]. Together these observations provide strong support for the importance of the inner centromere pool of Aurora B for error correction.

The spatial separation model explains the importance of the centromeric pool of Aurora B by suggesting that a continuous gradient of Aurora B activity extends from the sites of its enrichment at the inner centromere, while kinetochore substrates change their positions within this gradient depending on tension. Indeed, position changes of as little as 30–50 nm are associated with different levels of phosphorylation of both endogenous and exogenous Aurora B substrates at mammalian kinetochores [63,67]. How can such a steep gradient of kinase activity be established and maintained within the kinetochore, given that it is located hundreds of nanometers away from sites of Aurora B enrichment at the inner centromere? One idea links properties of the spatial activity gradient with the ability of Aurora B to activate itself via autophosphorylation, and to become conversely inactivated by a phosphatase [84,85,86,87,88]. Autoactivation in the context of a coupled kinase-phosphatase system leads to nonlinear, bistable behavior of kinase activity both in vitro and in cells, suggesting a physically plausible model for the formation of a steep kinase activity gradient [89]. In this model, Aurora B activates itself at the sites of highest concentration (i.e., inner centromere), overcoming inhibition by phosphatase. This activity then spreads throughout the chromatin thanks to a reaction-diffusion mechanism, in which active kinase molecules phosphorylate and activate additional kinase molecules that are either chromatin-bound or diffusing in the cytosol. As the Aurora B concentration decreases away from the inner centromere, phosphatase activity switches Aurora B to the inactive state in a highly nonlinear manner. Calculations suggest that this mechanism can potentially establish a steep gradient of kinase activity specifically in the kinetochore region, far away from the sites of highest Aurora B concentration [89].

**Figure 6:**
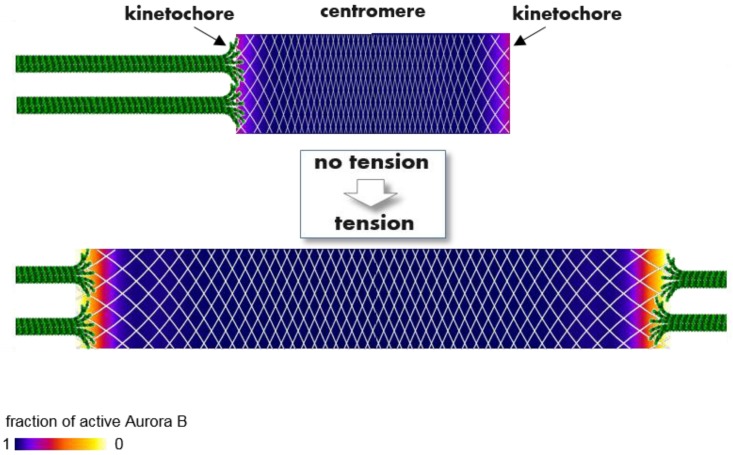
A molecular model to explain how tension regulates phosphorylation of kinetochore proteins that bind KMTs and regulate their attachment lifetime. Color-coded plots show the spatial distribution of active Aurora B kinase within a continuous flexible matrix (white mesh), encompassing the centromeric chromatin and two sister kinetochores (based on the theoretical model in [89]). Aurora B kinase is enriched strongly in the middle of the centromere (not shown), where it becomes highly active (purple and blue colors) due to trans-molecular auto-phosphorylation. With no tension this activity propagates from the centromere throughout the entire matrix, as active kinase “ignites” the nearby kinase, overcoming opposing phosphatases. As a result, kinase activity at the kinetochores is high with no tension, reducing KMT lifetime. When amphitelic KMTs stretch the connecting matrix, local Aurora B concentration is reduced everywhere (shown by increased spacing of the white mesh). However, the local concentration of active kinase does not decrease proportionally owing to the highly nonlinear, bistable nature of the underlying kinase-phosphatase switch. Aurora B kinase activity remains high within centromeric heterochromatin but drops sharply at the outer kinetochore (orange/red colors), forming phosphorylation gradients within the kinetochores. While this model provides a biophysical explanation for tension-dependent, long-range regulation of Aurora B kinase activity, the physiological significance of the kinetochore activity gradient seen in mammalian cells remains to be understood.

This hypothesis is additionally interesting because it implies that the centromeric chromatin and the kinetochore should be viewed as a continuous mechano-chemical medium. Stretching of this medium, which takes place when sister kinetochores are amphitelic, changes its biochemical properties due to changes in local Aurora B concentration everywhere within this medium (Figure 6). Because the coupled kinase-phosphatase system linked to this stretchable mechanical matrix is bistable, tension can exert strong and accurate control over the position and steepness of the Aurora B activity gradient at the kinetochore. This and other possible models for long-range effects of the centromeric Aurora B pool await critical examination. 

#### 2.4.4. Alternative Models

Although there is strong evidence that Aurora B function in regulating kinetochore-MT interactions depends on its targeting to the inner centromere in both fission yeast and mammalian cells, the situation in budding yeast is different. Removal of the normal centromere pool of Aurora B through mutation of its binding partner, INCENP, does not have major consequences for chromosome segregation or mitotic progression in budding yeast [90]. The reasons for this discrepancy are unclear and may reflect differences in Aurora B function between budding yeast and other systems that have been examined. For example, budding yeast is unusual in that each kinetochore binds only a single MT, and therefore there is no need to correct merotelic attachment errors, which may reduce the burden on Aurora B in destabilizing such attachments.

Alternatives to the spatial separation model, which could be applied to all organisms, not just yeast, assign no specific regulatory role to the centromeric Aurora B pool, and explain its importance for chromosome segregation by some other function [90]. For tension-dependent error correction, some models invoke a specialized pool of the kinase localized at kinetochores, in close proximity to its outer kinetochore targets. In one such model, stretching within the kinetochore itself separates Aurora B from its substrates, and an elongated INCENP subunit of the CPC may act as a flexible arm that determines how far the kinase can reach from its kinetochore binding site [68,91]. This model could explain how attachments are selectively stabilized, but only if stretching within the kinetochore correlates with tension on bi-oriented sister kinetochores, which does not appear to be the case [92,93,94]. Another model proposes a tension-sensitive binding site for Aurora B at the kinetochore [90]. In this case, increased tension would lead to a loss of kinase binding and stabilization of KMT attachments. It has also been proposed that Aurora B substrates are somehow inaccessible to the kinase when tension is high [69]. Yet another possibility is that phosphatase activity rather than kinase activity is regulated in response to tension, as a mechanism to control phosphorylation status of Aurora B substrates. Aurora B activity reduces kinetochore recruitment of protein phosphatase 1 (PP1) [95,96], but there is no evidence that phosphatase activity is directly regulated by tension. Because neither kinetochore binding sites for Aurora B nor tension-sensitive substrates have yet been identified, these different models are difficult to evaluate, and testing them is a challenging future goal.

#### 2.4.5. Selectivity of Aurora B-Dependent Error-Correction Mechanisms

Thus far, we have considered tension-dependent and tension-independent error-correction models in the frame of the simplest hypothesis that the underlying regulatory mechanisms are indiscriminate, i.e., at one kinetochore they effect the stability of correct and incorrect KMTs similarly. However, current views on Aurora B-dependent error correction often assume that modulation of KMT stability is selective, and the mechanism applies only to incorrectly attached MTs, while properly attached MTs at the same kinetochore are not regulated or are regulated oppositely [97]. It is not clear how this discrimination would work molecularly, and the mechanisms could be different for syntelic and merotelic errors.

Correction of syntelic attachments in yeast has been proposed to require that Aurora B specifically destabilizes the end-on attached syntelic MTs, while lateral MT attachments are resistant to its activity [98]. In this way Aurora B could promote repeated trial-and-error cycles until the tension-producing configuration is found and stabilized (Figure 1). Because amphitelic yeast KMTs are highly stable and do not turn over [99], Aurora B-dependent destabilization at this stage may not be needed.

With merotelic attachments that are common in cells with multiple KMTs, selectivity of a tension-dependent mechanism is difficult to explain because tension is shared by all MTs attached at the kinetochore, so it should affect them equally. One possibility is that regulation is dependent on the direction of tension, not just its magnitude. If merotelic KMTs are oriented more laterally to the kinetochore plane than amphitelic KMTs, their relative stabilities could be different [100]. Another model suggests that merotelic KMTs extend between the sister kinetochores; in this case, a centromere-localized kinesin-13 could selectively destabilize erroneous KMTs [101]. Yet another model builds on the observation that kinetochores with a bundle of merotelic KMTs are stretched such that part of the merotelic sister kinetochore extends towards the inner centromere, where Aurora B is enriched. In this case, kinetochore proteins bound to the plus-ends of incorrect KMTs may be closer to Aurora B and subject to increased kinase activity, while the ends of correct MTs would be unaffected [24,28,35,97]. Such strong kinetochore deformations are rarely seen, and it has been difficult to directly test this and other models because of the challenge of correlating differences in phosphorylation, MT end location and stability within a single kinetochore. 

Another model for how Aurora B destabilizes incorrect attachments is through regulation of kinase levels at the inner centromere. Aurora B is recruited to centromeres of chromosomes that are not properly aligned at the metaphase plate in multiple human cell lines [66,102], and at merotelic attachments in *Xenopus* cells [103]. This mechanism could act globally, i.e., affecting all KMTs at a merotelic kinetochore. For selective destabilization of merotelic KMTs it would have to rely on some signal to distinguish them from correct KMTs, but how such selectivity could be achieved remains unclear. Interestingly, a kinetochore configuration with merotelic KMTs has been found to persist longer than expected based on the measured metaphase KMT turnover rate [28,97]. This is in contrast with the idea of selective destabilization, which predicts that erroneous KMTs should be replaced faster. The continued presence of merotelic KMTs, however, could be explained by the basic error-correction mechanism, in which force from the merotelic KMTs prevents normal orientation of sister kinetochores [23]. On kinetochores that are not well oriented, the probability of binding wrong MTs is higher, so the merotelic configuration can persist. While future work may reveal a mechanism to selectively target incorrect vs. correct MTs at the same kinetochore, it is also possible that tension-dependent error-correction mechanisms regulate KMT turnover in a non-discriminating manner.

## 3. Conclusions

In cells with large number of kinetochore microtubules (KMTs), a surprisingly good accuracy of spindle microtubule (MT) attachments can potentially be achieved by the combined effects of back-to-back kinetochore geometry and indiscriminate KMT turnover. Optimal operation of this tension-independent error-correction mechanism requires expedient chromosome congression and moderate modulation of kinetochore geometry and turnover rate. These crucial properties are constrained by competing physiological requirements, such as formation of fully sized kinetochore fibers, and the speed and accuracy of segregation. The basic correction mechanism operates in combination with tension-induced stabilization of the amphitelic KMT configuration. Compelling evidence supports the essential role of the tension-dependent mechanism, especially for correcting syntelic attachments. While many questions remain about operation of this mechanism for correcting merotelic attachments, several molecular components that link tension and KMT stability, such as Aurora B kinase, have been identified and are under investigation.
